# IV Colistin: A Rare Cause of Bartter-Like Syndrome in Adults

**DOI:** 10.7759/cureus.50672

**Published:** 2023-12-17

**Authors:** Saiesh Dessai, Hemant Deshpande

**Affiliations:** 1 Department of Anaesthesiology, Jawaharlal Nehru Medical College, Datta Meghe Institute of Higher Education & Research, Wardha, IND

**Keywords:** bartter syndrome, transient bartter syndrome, antibiotic side effects, metabolic acidosis, colistin, critical care, hypokalaemia, bartter like syndrome

## Abstract

Bartter syndrome is a genetic condition characterized by autosomal recessive inheritance, resulting in impaired salt reabsorption and clinical manifestations such as low/normal blood pressure and extracellular fluid volume depletion. Multiple abnormalities of the electrolytes, including decreased potassium as well as chloride levels and, in some instances, hypomagnesemia, are its defining features. Metabolic alkalosis, hypokalaemia, hypocalcemia, and hypomagnesemia, together with adequate renal function, are all components of the Bartter-like syndrome. It is associated with certain antibiotics and antineoplastic drugs. We report a case of traumatic brain injury with pneumothorax who was on treatment on colistin and presented with metabolic disturbance.

## Introduction

Bartter syndrome or Bartter-like syndrome is a genetic condition characterized by autosomal recessive inheritance, resulting in impaired salt reabsorption and clinical manifestations such as low/normal blood pressure and extracellular fluid volume depletion [1]. Multiple abnormalities of the electrolytes, including decreased potassium and chloride levels and, in some instances, hypomagnesemia, are its defining features [2]. Antibiotics in the polymyxin class, also known as colistin, are polypeptides with strong Gram-negative activity first identified in 1947. The 1960s saw widespread usage of this class of drugs, but the 1970s and 1980s saw almost complete abolition of its use due to its negative effects. The emergence of multi-drug resistant pathogens, such as Klebsiella pneumoniae and Pseudomonas aeruginosa, is becoming increasingly prevalent, which has led to an increase in the use of polymyxins group of drugs as potential therapeutic agents during the past ten years [3]. One of the side effects associated with the use of colistin is characterized by renal damage and abrupt renal failure, which is seen to be the main drawback of this family of drugs. However, reports of other colistin toxicity, such as neurological problems, have been made [4,5]. Fewer cases of colistin-mediated neurotoxicity than renal damage have been reported in the literature, and it is characterized by a variety of symptoms, including facial and peripheral paraesthesia, ophthalmoplegia, trouble swallowing, vertigo, and respiratory apnoea [5].

## Case presentation

**Figure 1:**
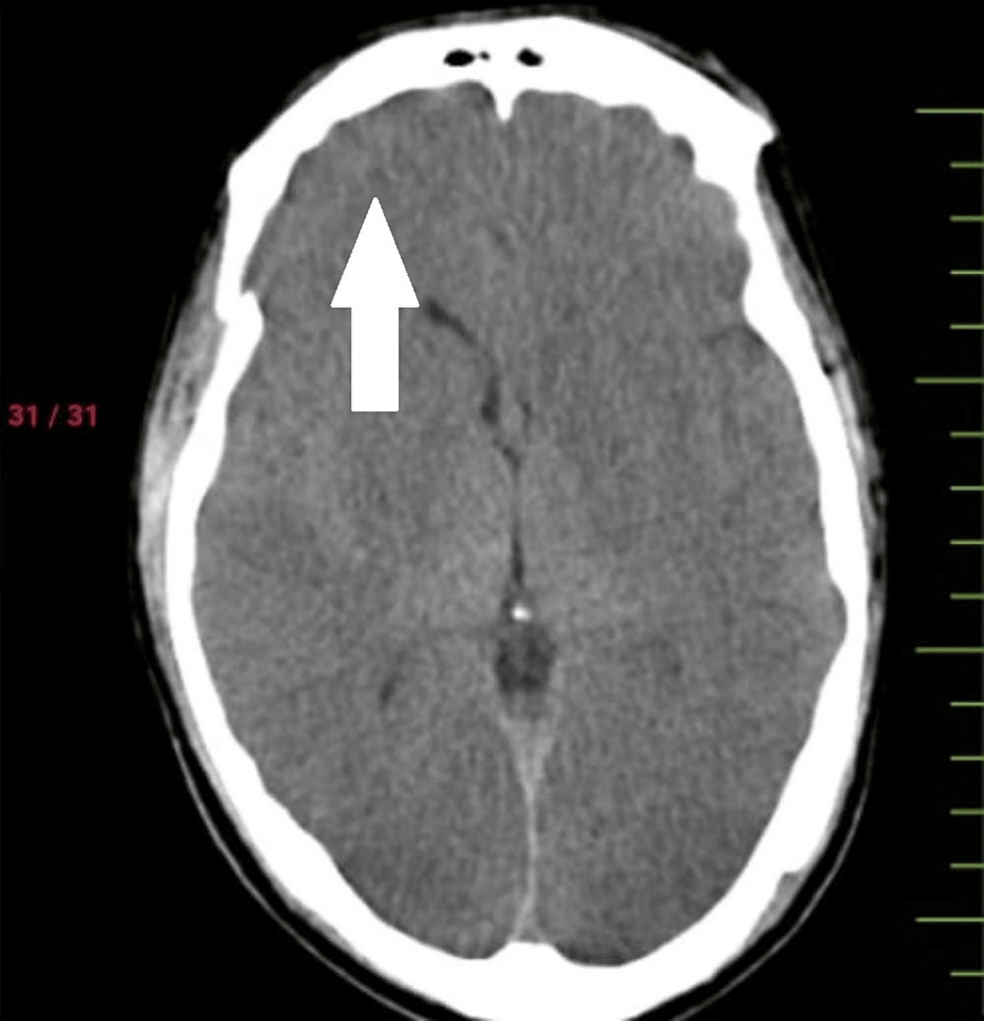
Pneumocephalus with subdural hemorrhage in the right frontal, basi-frontal, and tentorium cerebelli.

**Figure 2:**
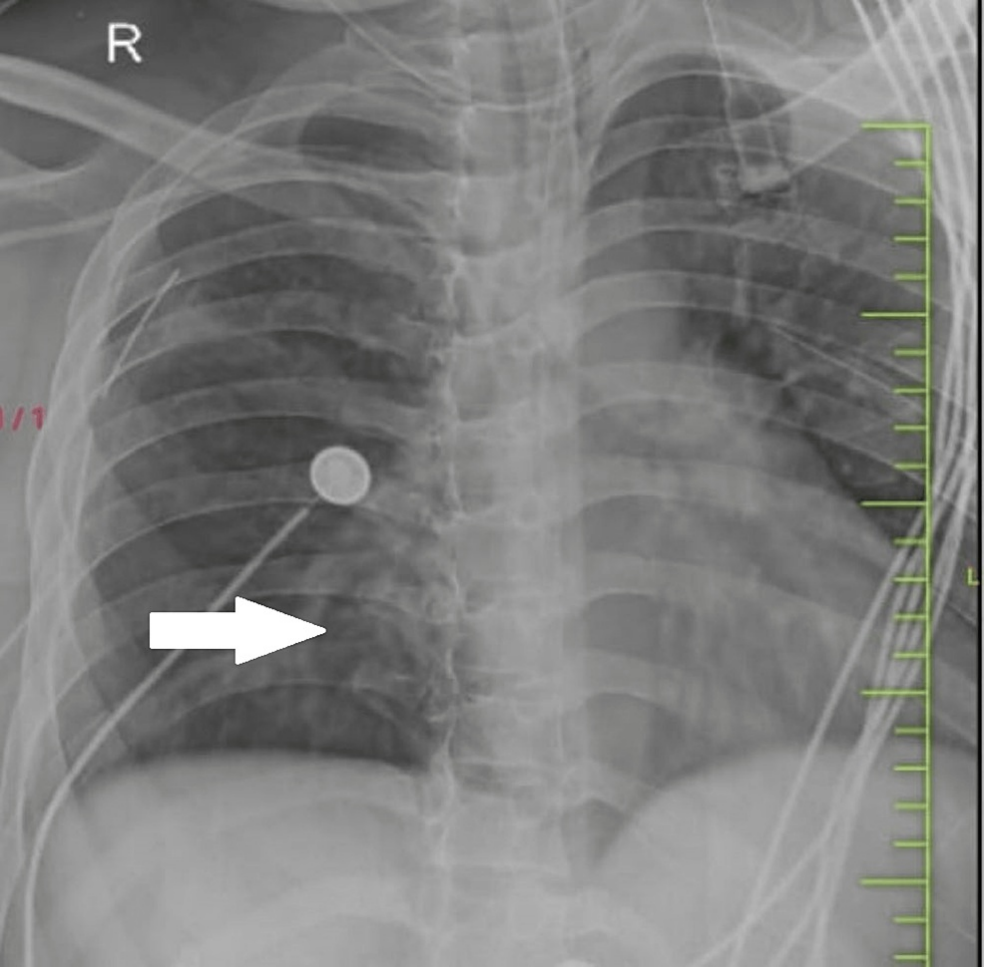
Bilateral pneumothorax with passive atelectasis with pneumopericardium and pneumomediastinum.

A 32-year-old man presented in casualty with a history of traumatic brain injury along with blunt chest trauma following an alleged history of trauma at the workplace. There was an associated history of ear bleeding present along with loss of consciousness. On arrival at casualty, he was immediately intubated because of a fall in consciousness. CT scan showed pneumocephalus with subdural hemorrhage in the right frontal, basi-frontal, and tentorium cerebelli (Figure 1), subarachnoid hemorrhage in an ambient cistern with intraventricular hemorrhage, along with multiple facial fractures, and right temporal fracture. The airway was secured with an endotracheal tube. His heart rate was 90 beats per minute, and his blood pressure was 130/80 mm Hg. There was a sudden episode of hypotension followed by bradycardia with a fall in saturation. Air entry was severely reduced bilaterally. A needle thoracotomy was done, and a hissing sound was noted. A bilaterally intercostal drain was inserted. CT showed signs of bilateral pneumothorax with passive atelectasis. Pneumocardium and pneumomediastinum were noted (Figure 2).

The cardiovascular surgeon advised no active management. The patient was started on cefoperazone with sulbactam 1.5 gm, along with supportive management. Injection fentanyl infusion was started with vecuronium and midazolam. Six days post-admission, a fever spike was noted. Tracheostomy cultures showed growth of Klebsiella and Pseudomonas, which were sensitive to colistin and piperacillin-tazobactam. The patient was started empirically on colistin, meropenem, and metronidazole. Twelve days post-admission, the patient went into bradycardia and hypotension. Injection atropine was given, following which pulse was 124, blood pressure was 134/90, and sugar level was 117 mg/dl. Oxygen saturation was 100 %. Arterial blood gas showed high anion gap metabolic acidosis along with hypokalaemia, hypocalcemia, and normal chloride levels (Table 1). Metabolic correction was given along with potassium and calcium correction. After six hours, an arterial blood gas was taken again, and it revealed that alkalosis persisted despite daily potassium supplements and prolonged hypokalaemia. The electrocardiogram showed flat T waves. Vomiting, diarrhea, and other possible sources of potassium loss were excluded. Other electrolyte disturbances included hypomagnesemia and hypocalcemia, and treatment for these conditions was initiated. We utilized the standard algorithm for hypokalaemia to arrive at the differential diagnosis. The patient also had metabolic alkalosis. A limitation of the case is that urine electrolytes could not be investigated.

**Table 1:** Blood investigations.

Investigations	Values	Normal range
Haemoglobin	8.1 g/dl	12-16 g/dl
Total leucocyte count	16400/ microliters	4000-10000/ microliters
Platelet	6.13 x 10^9 ^/L	1.5-4.5 x 10^9 ^/L
Potassium	3.8 mmol/L	3.5-5.1 mmol/L
Calcium	7.3 mg/dl	8.4-10.2 mg/dl
Magnesium	3.4 mg/dl	1.6-2.3 mg/dl
Phosphorus	6.4 mg/dl	2.5-4.5 mg/dl
Chloride	101 mmol/L	98-107 mmol/L
pH	7.35	

## Discussion

Colistin belongs to a group of polymyxins that are derived from Bacillus polymyxa (B. polymyxa) [6]. It acts by disrupting the permeability of the cell membrane by displacing the magnesium and calcium that help retain anionic lipopolysaccharide component in the outermost cell membrane of Gram-negative bacteria leading to its bactericidal action [5,7]. Bartter syndrome is classified as classic, neonatal, and Gitelman syndrome. There are also rare cases of Bartter-like syndrome, which do not fit the criteria but share similar features, such as electrolyte imbalances and kidney dysfunction [2]. There are five different types of Bartter syndrome: I, II, III, IV, IVb, and V [8].

Nephrotoxicity and neurotoxicity are the side effects of colistin treatment that occur most frequently. One potential way colistin can indirectly lead to hypokalemia is through its effects on the kidneys. Colistin is primarily excreted by the kidneys, and in some cases, it can cause renal dysfunction or kidney damage, which may impair the kidneys' ability to regulate electrolytes, including potassium. This can potentially lead to electrolyte imbalances, including hypokalemia. Colistin-induced nephrotoxicity is frequently accompanied by a decrease in urine output along with elevated creatinine in serum [4]. Colistin may produce dose-dependent, generally reversible, acute renal damage that may necessitate hemodialysis [5]. Additionally, some individuals have nephrocalcinosis and hypercalciuria [9].

Colistin is usually used in cases where infections are resistant to preliminary antibiotics [10]. Patients who receive colistin may have other risk factors for developing hypokalemia, such as being on diuretic medications that can increase urinary excretion of potassium, having fluid imbalances, or having underlying health conditions that affect potassium metabolism. Our patient experienced prolonged colistin therapy with unexplained hypokalaemia, metabolic alkalosis, and other renal-related electrolyte abnormalities [11]. Milder cases often manifest later in childhood/adolescence or as adults, with polyuria, exhaustion, failure to thrive, and muscular cramps (e.g., Bartter syndrome kinds 3). Typically, serum potassium ranges between 3.5 to 5 mEq/L. However, imbalances in potassium levels can occur due to various factors, such as hormonal influences, diseases, and nutritional deficiencies, which can disrupt acid-base balance, aldosterone levels, insulin regulation, catecholamine levels, and body fluid tonicity [12]. The presence of hypomagnesemia in over half of all clinically relevant hypokalemia cases causes hypokalemia to develop by lowering intracellular potassium content [13]. It is a differential for metabolic alkalosis, which is more frequently found in those who use diuretics and have hypokalemia [14]. In Bartter syndrome, the afflicted segment has poor salt chloride reabsorption, which results in hypokalaemia, hypochloraemia, and metabolic alkalosis.

Acquired Bartter-like syndrome has also been reported, particularly in cases where aminoglycoside antibiotics have been used [8,9,15]. Treatment for Bartter syndrome typically involves managing the imbalances in electrolytes through medications and dietary adjustments. This may include medications to replace or regulate electrolytes, such as potassium or magnesium supplements, as well as medications to block the effects of certain hormones that affect electrolyte reabsorption. To maintain metabolic balance, aggressive IV electrolyte replacement, and IV hydration were essential daily [8]. The mechanism of toxicity is poorly understood, although in vitro electrophysiological experiments showed that colistin has harmful effects on urothelium at extended exposure durations by enhancing transepithelial conduction. There have been reports of probable abnormalities in the ascending loop of Henle caused by medications such as furosemide, cisplatin, carbenicillin, cyclosporine, amphotericin, and chronic administration of gentamicin, capreomycin, and streptomycin [4].

## Conclusions

In the continuous fight against microorganisms that are resistant to antibiotics, colistin usage is unavoidable and increasing. In addition to the main illness process and comorbidities, a combination of drugs and adverse antibiotic responses also have a significant role, as evidenced by hypokalaemia. When using colistin, electrolyte imbalances may arise with normal renal function, although they seem to get corrected when the medication is stopped. Measures should be taken to combat or control variables such as shock, hypoalbuminemia, and simultaneous use of potentially nephrotoxic medications that might worsen the nephrotoxicity of colistin. The risk of developing hypokalaemia from colistin should be considered and should be carefully weighed against the potential benefits of the antibiotic in treating specific bacterial infections. Close monitoring of electrolyte levels, including potassium and kidney function, is typically recommended during colistin therapy, especially in high-risk patients.
